# Chromato-panning: an efficient new mode of identifying suitable ligands from phage display libraries

**DOI:** 10.1186/1472-6750-9-21

**Published:** 2009-03-17

**Authors:** Wim Noppe, Fatima Plieva, Igor Yu Galaev, Hans Pottel, Hans Deckmyn, Bo Mattiasson

**Affiliations:** 1 Interdisciplinary Research Center, Katholieke Universiteit Leuven Campus Kortrijk, E Sabbelaan 53, B-8500 Kortrijk, Belgium; 2 Department of Biotechnology, Lund University, P. O. Box 124, SE-22100 Lund, Sweden; 3 Protista Biotechnology AB, P.O. Box 86, SE-26722 Lund, Sweden

## Abstract

**Background:**

Phage Display technology is a well established technique for high throughput screening of affinity ligands. Here we describe a new compact chromato-panning procedure for selection of suitable binders from a phage peptide display library.

**Results:**

Both phages and *E. coli *cells pass non-hindered through the interconnected pores of macroporous gel, so called *cryogel*. After coupling a ligand to a monolithic cryogel column, the phage library was applied on the column and non-bound phages were washed out. The selection of strong phage-binders was achieved already after the first panning cycle due to the efficient separation of phage-binders from phage-non-binders in chromatographic mode rather than in batch mode as in traditional biopanning procedures. *E. coli *cells were applied on the column for infection with the specifically bound phages.

**Conclusion:**

Chromato-panning allows combining several steps of the panning procedure resulting in 4–8 fold decrease of total time needed for phage selection.

## Background

Since the introduction of the phage display peptide libraries in 1985 [1], when a vector could directly display a small foreign peptide in one of the coating proteins of the filamentous M13 phage particle, the greatest task has been to find suitable ligands. Because of the need for repeated panning rounds to isolate high-affinity ligands, the whole panning procedure is rather laborious and time consuming. Therefore, a shorter, more efficient procedure, with fewer steps and fewer panning cycles would be desirable.

Phage display is suitable not only for peptides, but also for small proteins and protein fragments (*e.g*. antibody fragments), which can be expressed on the phage surface proteins [2]. Several linear [3-6] and constrained cyclic [7-10] phage peptide libraries have been sucessfully used to screen for affinity ligands against a variety of targets, all of which were based on use of the laborious, repetitive biopanning procedure. The technique of phage display and its applications is more extensively discussed in some recent review papers. C. Adda et al. [2] and J. Kehoe et al.[11] review the technique of phage display in all its aspects. M. Paschke [12]; M. Szardenings [13]; M. Arap [14]; C. Mersich et al. [15] and V. Petrenko [16] present extensive reviews on the present state and applications of the phage display technology.

**Figure 1:**
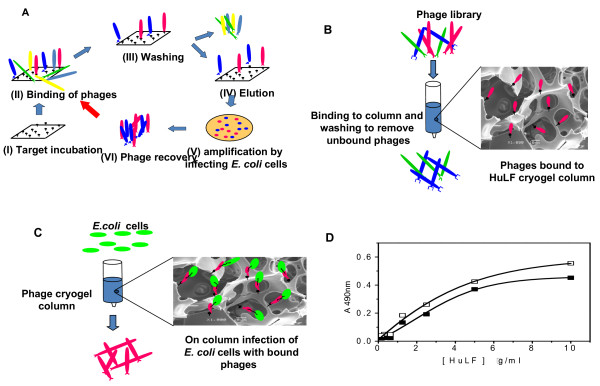
**Panning procedures**. (a) Schematic representation of a traditional biopanning procedure. After target incubation (I) and postcoating, a phage library is added and incubated (II). In a washing step (III) non-bound phages are washed away followed by an elution step (IV). The *E. coli *cells are consequently infected with the eluted phages (V) and out-plated on agar plates for amplification. After overnight grow the cells are harvested and the phages revovered by a precipitation step (VI). These phages are used for subsequent panning rounds until high affinity phages are obtained. Schematic representation of the Chromato-panning procedure: (b) On-column panning. A cryogel column with the target protein covalently coupled, is washed with PBS buffer followed by loading of a sample of the phage library at 0,5 ml.min^-1^. After washing out the non-bound phages, the bound phages can be eluted with 1 M NaCl and further used for infection of *E. coli *cells as described for a classical panning procedure. (c) On-column infection. After the on-column panning as in (b), without elution of bound phages, the column is incubated at 37°C and one column volume of *E. coli *cells is applied on the column, followed by an incubation time (no flow) for 45 min. Next, the infected *E. coli *cells are eluted and out-plated on agar plates. After overnight grow, the cells are harvested and the phages recovered by a precipitation step. (d) Evaluation of the elution solvent in biopanning. Two similar chromato-panning procedures were performed with a linear hexamer peptide library using a different elution solvent. The bound phages were eluted from the column using either 0.1 M glycine, pH 2.2 (open square) or 1 M NaCl (black square) as elution solvent. The eluted phages were used to infect *E. coli *cells. After amplification and harvesting, the binding affinity of the harvested phages was analyzed using phage ELISA as described in Materials and Methods.

To separate phages binding to the ligand from non-binders, the ligand is immobilized on a solid carrier allowing for easy separation of the liquid phase, which contains predominantly non-binding phages, from the solid phase, which contains mainly binding phages (Figure 1a). Many variations of the technique have been described, however, rigid plastic materials, such as polystyrene, have traditionally been used as carriers to provide a nonporous interface for ligand immobilization. As the diffusivity of phages is negligible due to their size, the ligands immobilized in diffusional pores will be essentially unavailable to the phages. To provide a larger interface area, the size of the carrier beads can be decreased or the surface can be decorated with tentacles. However, this does not circumvent the main shortcoming of the traditional biopanning process, namely that washing is carried out in batch mode which is a "one-plate" operation and hence rather inefficient from a bioseparation point of view. Not surprisingly, several repetitive rounds (*i.e*. a few "one-plate" operations) are needed to isolate strong binders. The separation of a liquid phase from a solid phase in chromatographic mode (multi-plate operation) could be assumed to be much more efficient, giving efficient separation of binders from non-binders in a single operation. To determine whether this is in fact the case, an affinity chromatographic material that combines several non-trivial characteristics is needed. Such a material should:

(i) be macroporous, with a pore size no less than 10 μm, as the phages themselves are about 1 μm long (larger pore sizes, up to 100 μm, are desirable to allow the possibility of direct infection of the cells by the phages bound to the affinity matrix);

(ii) have highly interconnected pores, allowing convectional rather than diffusional mass transport of phages and presumably cells inside the pores;

(iii) be hydrophilic with minimal non-specific binding of phages or cells and hence no need for post-coating;

(iv) contain active groups for the immobilization of the ligand.

We have recently presented a material with the required combination of properties for chromatographic separation of microbial and mammalian cells [17-20]. Macroporous hydrogels, or *cryogels*, are produced using polymerization in semi-frozen conditions, where ice crystals perform the role of the porogen, and polymerization proceeds in the non-frozen liquid phase [21]. The properties of cryogels and the architecture of interconnected macropores are presented in detail in recent publications by Plieva et al. [22-24]. In this communication we present a new chromato-panning procedure based on using a cryogel as a matrix for ligand immobilization.

## Results and discussion

### On-column panning

**Figure 2:**
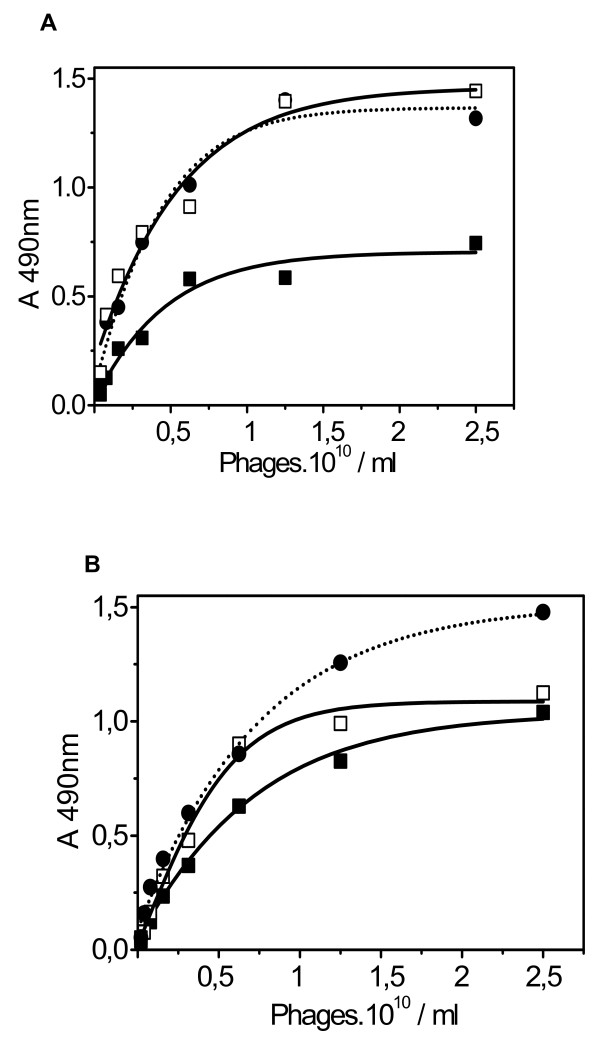
**Efficiency of the panning procedures**. Different phage libraries, C6 and L15, were used in both a tube biopanning and a chromato-panning procedure. In both procedures three rounds were performed as described in Table 1. The binding efficiency of the obtained phages was determined using HuLF ELISA as described in Materials and Methods. (**a**) Biopanning with a C6 library. Tube panning: Round 1 (black square) Round 3 (open square); column panning: Round 1 (black circle-dotted line). (**b**) Biopanning with a L15 library. Tube panning: Round 1 (black square) and Round 3 (open square); column panning: Round 1 (black circle-dotted line).

The efficiency of the column panning procedure (Figure 1b) was studied by comparing the performance of the chromato-panning procedure with that of the tube panning procedure. Different phage libraries were used, and three panning rounds were performed. Human lactoferrin (HuLF) was used as the ligand, and this was coupled to the column as described in Materials and Methods. The most frequently used elution media in biopanning procedures are glycine and hydrochloric acid at a pH of ~2.2. Frequent use of these acidic buffers may denature the bound ligand, leading to a loss in binding capacity. In earlier work [25,26] 1 M NaCl was used as elution medium to elute protein from a cryogel-phage column with good results. To evaluate the elution strength both elution media were investigated in a column panning procedure, showing similar elution efficiencies (Figure 1d). Based on these findings both elution solvents were used in further experiments: (i) for the tube panning procedure, 0.1 M glycine, pH 2.2, was used as the elution medium and (ii) for the column panning procedure 1 M NaCl was used as elution medium. After elution, phages from each panning round were amplified and recovered, their binding efficiencies were tested using a phage ELISA as described in Materials and Methods. Different phage libraries, a cyclic hexamer (C6), linear hexamer (L6) and a linear pentadecamer (L15) library, were used for biopanning. After one round in the column panning procedure, the selected phages show comparable (C6 library) or somewhat higher (L15 library) binding strength towards HuLF as compared to the phages obtained after the third panning round in the tube panning experiment (Figure 2a, b). The L6 library showed similar results to the C6 library (data not shown). In the tube panning experiment an increase in binding strength was observed in consecutive panning rounds (R3 > R2 > R1). In the column panning procedure, no further increase was observed after the first round (L6 and C6 library). Table 1. shows that a statistical significant difference (p < 0.05) between top-values (plateau levels) is observed for all three used between the first round of the tube panning and the chromato-panning procedure, which disappeared after the third tube panning round. Furthermore, also a paired comparison over the three libraries between the first round of the tube panning and the chromato-panning procedure resulted in a statistically significant difference (p = 0.025). These results show the superiority of the chromato-panning procedure for fast screening for high affinity ligands.

**Table 1:** Statistical analysis data

Library	Id	Panning round	Top-value	95% CI	R^2^
C6	a	T r1	0.853	[0.712–0.994]^bc^	0.979
	b	T r3	1.622	[1.382–1.862]^a^	0.978
	c	C r1	1.605	[1.370–1.839]^a^	0.981

L6	a	T r1	0.697	[0.600–0.794]^bc^	0.990
	b	T r3	1.640	[1.500–1.780]^a^	0.990
	c	C r1	1.545	[1.299–1.790]^a^	0.962

L15	a	T r1	1.354	[1.279–1.430]^c^	0.999
	b	T r3	1.382	[1.180–1.584]^c^	0.986
	c	C r1	1.832	[1.645–2.020]^ab^	0.994

For the three phage libraries C6, L6 and L15, top-values, 95% CI and R^2 ^(see Material and Methods) was calculated and compared for the tube panning round 1, 3 (Tr1, Tr3) and chromato-panning procedure round 3 (Cr1). Superscripts denote statistical significant difference (p < 0.05) between top-values (e.g. top "a" is statistically significant different from tops "b" and "c").

### On-column infection

To further optimize the column panning procedure, on-column infection of *E. coli *cells was investigated. The macropores in the cryogel are large enough to allow *E. coli *cells to pass freely through the plain cryogel without blocking the pores [26]. However, on the HuLF cryogel we observed binding of cells that could be eluted from the cryogel with NaCl, in accordance with previous observations that *E. coli *cells bound to an anion-exchange cryogel column were eluted by NaCl [27]. As HuLF is positively charged at pH ~7.0, the HuLF-cryogel may act as an "anion-exchanger", which could explain our observation. NaCl (1 M) eluted all cells from the column. By immediately diluting the 1 M NaCl eluate in Luria broth (LB) medium, the final NaCl concentration of the eluted cell suspension was reduced to approx. 0.4 M NaCl. This NaCl concentration had no negative effect on the viability of the cells. Under these conditions we observed a similar growth rate on agar plates to that of cells that had not come into contact with high salt concentrations.

**Figure 3:**
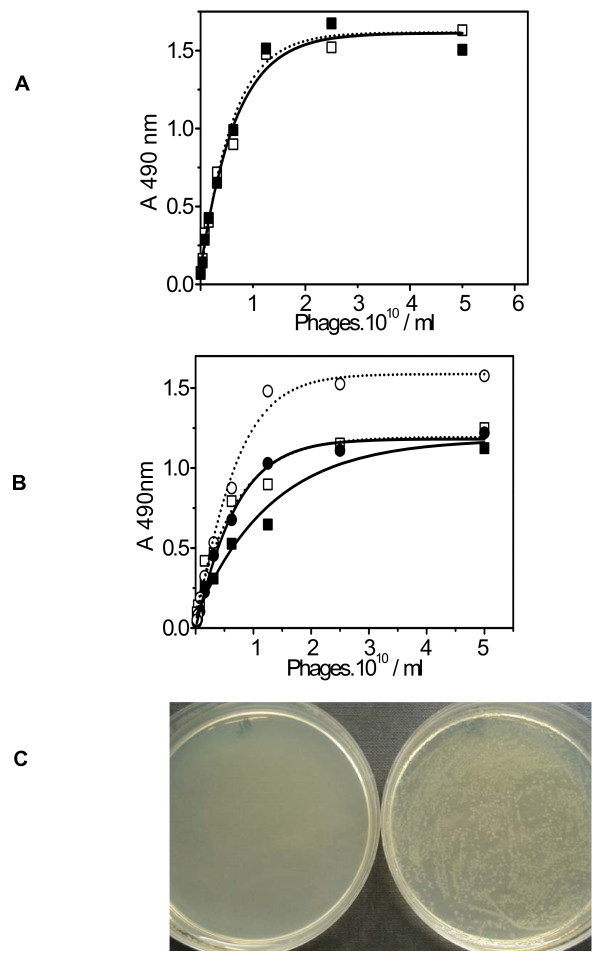
**On-column *E. coli *infection**. Two phage libraries, L6 and L15, were used for column panning and on-column infection. Two rounds were performed as described in Table 1. As a reference sample, a single phage clone HuN, was used for on-column infection. The binding efficiency was determined using HuLF ELISA as described in Material and Methods. (**a**) On-column cell infection on a HuLF column with the HuN single-phage clone. HuN phage reference sample (no infection performed) (black square) and HuN fraction eluted with 1 M NaCl after on-column infection (open square-dotted line). (**b**) Column panning and on-column cell infection on the HuLF column with the L6 and L15 phage peptide libraries. Two panning rounds were performed. Phages from the L6 phage peptide library (solid lines): Round 1 (black square) and Round 2 (open square); phages from the L15 phage peptide library (dotted lines): Round 1 (black circle) and Round 2 (open circle ○). (**c**) Control of cell growth on agar plates after on-column phage infection. (left): *E. coli *cells which were not infected with phages were plated on agar plates containing tetracycline and incubated overnight at 37°C. (right): *E. coli *cells were applied to the HuLF column on which phages were bound after a panning round. After cell infection and elution, the 1 M NaCl fraction was plated on agar plates containing tetracycline and grown overnight at 37°C.

The on-column infection procedure was first tested with a single-clone phage (HuN phage clone) as described in Materials and Methods. The amount of amplified phages after on-column infection was comparable to the amount obtained in a tube panning procedure or a column panning procedure with a separate infection step. The binding strength of the phages obtained was similar to that for phages obtained from a normal panning procedure (Figure 3a). A similar result was obtained with phage clones Hu14 and Hu5 (data not shown) although the three phage clones expressed a different peptide sequence on the pIII protein [7]. The same procedure was consequently performed with the L6 and L15 phage libraries. Two column panning rounds and on-column infection were performed with each phage library. The amount of phages and the binding strength were determined after each panning round. Again, the amount of phages was comparable to the amount of phages obtained in the other panning procedures. The binding strength was also similar as that obtained in the column panning procedure with a separate infection step. Again, the results show that only one panning round is necessary to obtain specific phages for the L6 library. Statistical analysis shows no significant difference between the top-values of round 1 and 2 of the chromato-panning (95% CI round 1: 1.069–1.432; 95% CI round 2: 1.199–1.393). For the L15 library still some increase is observed after the second round (95% CI round 1: 1.295–1.550; 95% CI round 2: 1.627–2.176), probably due to the higher diversity of phages present in the library (Figure 3b), indicating that a second panning round might here be advisable. These results are in agreement with those shown in Figure 2b, where a similar column panning procedure was performed except that the infection step was performed separately and not on-column. These results confirm that biopanning was achieved on the HuLF column, and that on-column infection of the *E. coli *cells took place.

To further confirm the on-column infection of the *E. coli *cells we measured the amplification rate of the *E. coli *cells after infection on the column. *E. coli *cells that were not in contact with phages (blank sample) did not grow on agar plates containing tetracycline. *E. coli *cells that were infected by phages on the column survived and grew on the agar plates containing tetracycline (Figure 3c). After the cells were infected and eluted from the column, the cell suspension was centrifuged and the pellet re-suspended in LB buffer. At this point, the optical density at 600 nm (OD^600^) was measured. After plating, overnight incubation at 37°C and harvesting the cells in LB medium, OD^600 ^nm was measured again. A ~950 fold increase in OD^600 ^was observed, which indicates that the cells were effectively infected on the HuLF cryogel column. The new panning procedure is independent of the ligand or phage library used. We used different phage peptide libraries and two different proteins as the ligand. The results presented above are based on results obtained with HuLF as ligand. Similar results were obtained in an experiment with α-chymotrypsin as the ligand and the L6 phage peptide library (data not shown).

### Chromato-panning procedure

To evaluate the chromato-panning procedure it was compared with the traditional biopanning procedure (Table 2) as well with other protocols (Table 3) described in the literature [28-30]. Special attention was paid to the total time required for the procedure, the efficiency of the elution media, and the efficiency of the procedure in terms of the number of rounds necessary to obtain high-affinity binders.

**Table 2:** Comparative time frame of chromato-panning protocol and tube panning protocol

Chromato-panning		Tube panning	
Round 1 (h)		Round 1 (h)	

Column equilibration	0.25	Target coating	18
		Wash-postcoat	2.25
On column panning	1	Phage incubation	20
		Washing/elution	0.50
On column infection	0.50	Infection	0.50
Elution	0.25		
Plating/amplification	18	Plating/amplification	18
Phage recovery	3	Phage recovery	3
			

**Time**	**~23 h**		~**62 h**

No additional panning rounds are needed		2 additional panning rounds are performed Phage incubation R2/3	2.50
			
**TOTAL TIME**	**~23 h**		~**152 h**

A mean time frame of each step in the panning procedure is presented for one panning round. The total time represents the time frame for a complete panning procedure as described in Methods.

**Table 3:** Comparative time frames of reported panning protocols

Microtiter plate [28]		Microtiter plate [29]		RISE [30]	
Round 1 (h)		Round 1 (h)		Round 1 (h)	

Target incubation	18	Target incubation	2	Target incubation	18
Wash-postcoat	2.25	Wash-postcoat	1.5	Wash-postcoat	4
Phage incubation	1	Phage incubation	1	Cell growth tubes	3
Wash-elution	0.5	Wash-elution	2.25	Infection	3
Infection		Infection		Wash	0.5
- amplification	4.5	- amplification	5	Cell/phage/target incubation	16
Phage recovery	0.5	Phage recovery	18	Phage recovery	0.5
					

**Time**	**~27 h**		**~30 h**		**~45 h**

2 additional panning rounds		2 additional panning rounds		3 additional panning rounds	
					
**TOTAL TIME**	**~81 h**		**~90 h**		**~180 h**

A mean time frame of each step in the panning procedure is presented for one panning round. The total time represents the time frame for a complete panning procedure as reported in the literature.

The total time is considerably shorter when using a column procedure (Table 2). Advantages of the column procedure are: no target coating is needed for each round as the column is re-usable, no post-coating is needed, the contact time between the phage-containing solution and the column is shorter and, finally, a milder elution medium can be used. The first column panning round takes about 1/3 of the time required for the first round in a tube panning procedure. If three rounds were needed a reduction of approximately 50% in time is obtained. Only 1 round was necessary to obtain high-affinity binders in the column panning procedure, compared to 3 rounds in the tube panning procedure. In this case column panning takes only approximately 1/6 of the time needed for a tube panning.

## Conclusion

The use of macroporous cryogels in a chromato-panning procedure has various advantages. The large interconnecting pores of the cryogel allow (i) free passage of bacteriophages during the panning step without clogging the column, and (ii) free passage of *E. coli *cells so that infection can take place on-column. (iii) A significant time saving can be achieved as only one column panning round is needed to select high-affinity phage clones. (iv) A milder elution solvent can be used to elute phages and *E. coli *cells, without denaturing the bound ligand on the column or having negative effects on the viability of the cells. At present we provided "proof-of-principle", it is clear that our procedure as well as the data need to be more extensively validated in the future before the method can be broadly used as an alternative for the classical biopanning and as a new platform for rapid screening of ligands.

## Methods

### Preparation of cryogel monoliths

The epoxy-activated macroporous cryogels were produced and provided by Protista Biotechnology AB (Lund, Sweden) according to published protocols [17].

### Tube biopanning procedure

A solution of human lactoferrin (HuLF) at 10 μg/ml in phosphate-buffered saline (PBS) was coated for 2 hours at room temperature (RT) on sterile Nunc immunotubes (Nunc A/S) placed on a test-tube rotator at 10 rpm, followed by standing incubation overnight at 4°C. (Figure 1a). After discarding the coating solution the tubes were washed with PBS containing 0.2% Tween-20 (TPBS). Post-coating was performed with a 4% (wt/vol) solution of milk powder in PBS for at least 2 hours at RT. After washing with TPBS, 2 × 10^12 ^phages from a phage-display peptide library were added in PBS to the tube and incubated overnight at 4°C on a test-tube rotator at 10 rpm. After careful washing with TPBS, the bound phages were eluted with 0.1 M glycine, pH 2.0, on a test-tube rotator for 15 min at RT. The eluate was neutralized by adding 1 M Tris pH 8.0 and kept on ice until infection. An overnight *E. coli *TG-1 (Stratagene) cell culture was diluted 100 times and grown at 37°C on a shaker until the OD^600 ^was ~0.500. To 650 μl eluted phages, 10 ml of the *E. coli *TG-1 cell culture was added and incubated at 37°C for phage infection. After incubation, the cell suspension was centrifuged and the cell pellet was re-suspended in sterile Luria broth (LB) medium (Invitrogen). The cell suspension was subsequently plated on Luria agar plates containing 20 μg/ml tetracycline-hydrochloride, and incubated overnight at 37°C.

The cells were recovered in sterile LB medium and the cell suspension was centrifuged for 20 min at 7600 × g at 4°C. To the clear supernatant, 1/3 to 1/5 volume of 20% PEG-6000/2.5 M NaCl was added, mixed and incubated on ice for 90 min for phage precipitation. Phages were recovered by centrifugation at 2000 × g for 20 min. The pellet was re-suspended in sterile distilled water and precipitated again with the PEG/NaCl solution. After incubation and centrifugation the phage pellet was finally re-suspended in PBS. The phage concentration was measured as the absorbance at 260 nm (A^260^) (1A unit = 2.2 × 10^11 ^phages.ml^-1^).

In order to obtain phages specific to the ligand, three panning rounds were performed. In the consecutive panning rounds, 1 × 10^11 ^phages of the previous panning round was used for incubation in the coated immunotube. The incubation time was reduced to 90 min on a test-tube rotator followed by standing incubation for 60 min. All ensuing steps were similar to those described for the first panning round. Finally, the phages were stored in PBS buffer – 5% (wt/vol) glycerol at -20°C until further use. All buffers were filtered through a 0.22 μm filter and autoclaved before use.

### Ligand coupling to the monolithic cryogel

Two dried epoxy-activated monolithic cryogels were inserted, one on top of the other, in a chromatographic column. The monolithic cryogels were swollen in sterile water and the upper adaptor was lowered onto the gel surface (9 ml bed volume). The column was washed with sterile water and equilibrated with 0.1 M carbonate buffer, pH 9.5, at a flow rate of 1 ml/min. A solution of HuLF in 0.1 M carbonate buffer, pH 9.5, was re-circulated over the column for 16 hours at a flow rate of 0.5 ml/min at 4°C. After coupling, the column was washed with 0.1 M carbonate buffer, pH 9.5, until all non-bound HuLF had been removed, followed by re-circulation of 25 ml 0.1 M ethanolamine in 0.1 M carbonate buffer, pH 9.5, for 3 hours at a flow rate of 1 ml/min to block the remaining active epoxy groups. Finally, the column was washed with sterile water and PBS, and stored at 4°C until further use.

### Chromato-panning

The HuLF-cryogel column (Figure 1b) was washed with sterile PBS. Two ml of solution containing 1 × 10^12 ^phages of a phage-displayed peptide library was loaded onto the column at 0.5 ml.min^-1^, followed by washing with 30 ml PBS to remove unbound phages. The bound phage fraction was eluted, 1 ml fractions were collected and stored at -20°C until further use. Two different elution media were investigated: 0.1 M glycine, pH 2.0 (used in the tube panning procedure), and 1 M NaCl. After elution the column was washed with 2 M NaCl and re-equilibrated with PBS buffer. The eluted phages were amplified by infecting *E. coli *TG-1 cells as described above. Three panning rounds were performed using the same HuLF cryogel column.

### On-column cell infection

The chromato-panning procedure was very similar to that described above. Instead of eluting the phages from the column, *E. coli *TG-1 cells were loaded onto the column for infection and phage amplification (Figure 1c). The phage-displayed peptide library was loaded onto the column, which was then washed with PBS to remove unbound phages. The column was then incubated at 37°C. An overnight *E. coli *TG-1 cell culture was diluted 100 times and grown at 37°C on a shaker until OD^600 ^reached ~0.5. One column volume, approx. 8–9 ml cell suspension, was loaded onto the column and the flow was stopped. The cells were incubated on the column for 30 min at 37°C. The cells were then eluted from the column by washing with 20 ml LB medium containing 1 M NaCl. The eluate was collected in 20 ml LB medium to dilute the NaCl in order to maintain the viability of the *E. coli *cells. The cell suspension was immediately centrifuged at 750 × g for 10 min. The pellet was re-suspended in LB medium and plated on Luria agar plates containing 20 μg/ml tetracycline-hydrochloride, and incubated overnight at 37°C. Phage recovery was similar to that described above. A second round of chromato-panning and on-column infection was performed in a similar way. In the second round the phage concentration was reduced to 5 × 10^10 ^phages.ml^-1 ^(2 ml applied to the column). All buffers used had been filtered through a 0.22 μm filter and autoclaved. All procedures were performed at room temperature, except for the on-column infection and cell/phage elution which were performed at 37°C.

### Human lactoferrin ELISA

The wells of a Microlon ELISA plate (Greiner Bio-One) were coated with 10 μg/ml of HuLF in PBS buffer and left overnight at 4°C. After washing with TPBS, the wells were post-coated with a 4% (wt/vol) solution of milk powder in PBS for at least 2 hours at RT. After washing, a 2-fold serial dilution of phages starting at 1 × 10^11 ^phages/ml in 0.4% (wt/vol) milk powder in PBS was added to the wells and incubated for 2 hours at RT. After extensive washing with TPBS, HRP/anti-M13 monoclonal conjugate (GE Healthcare) (1:2500 in 0.4% (wt/vol) milk powder in PBS) was added and incubated for 1 hour at RT. After extensive washing, *o*-phenylenediamine hydrochloride and hydrogen peroxide solution was added. The reaction was stopped by adding 4 M sulfuric acid and the absorbance was measured at 490 nm using a BIO-Tek ELISA reader.

### Phage ELISA

The wells of a Microlon ELISA plate were coated with 100 μl of 5 × 10^10 ^phages.ml^-1 ^in PBS buffer overnight at 4°C. After washing with TPBS, the wells were post-coated with a 4% (wt/vol) solution of milk powder in PBS for at least 2 hours at RT. After washing, a 2-fold serial dilution HuLF at 10 μg/ml in 0.4% (wt/vol) milk powder in PBS was added to the wells and incubated for 2 hours at RT. After extensive washing with TPBS, HRP/Goat anti-HuLF conjugate (Nordic) (1:5000 in 0.4% (wt/vol) milk powder in PBS) was added and incubated for 1 hour at RT. After extensive washing, *o*-phenylenediamine hydrochloride and hydrogen peroxide solution was added. The reaction was stopped by adding 4 M sulfuric acid and the absorbance was measured at 490 nm using a BIO-Tek ELISA reader.

### Statistical analysis

The data (absorbance versus phages) for the three libraries (C6, L6 and L15) were fitted with a simple sigmoidal dose-response model, starting from Abs = 0 and ending at Abs = Top: Abs = Top/[1 + 10^(logEC50 - X)^], with X the log-transformed concentration of phages/ml (GraphPad Prism 5.0). The fit parameter logEC50 corresponds to the concentration of phages at half the saturation level. The fit parameter Top, along with its 95% Confidence Interval (CI), is used to represent the saturation level of the measured absorbance (for X → ∞). Statistical difference between saturation levels for tube panning after round 1 (R1) versus chromato-panning R1 for each library was evaluated based on non-overlapping 95% CI's (corresponding to p < 0.05). The good quality of the fits can be judged from the R^2^-value (explained variance).

An overall judgment of statistical difference for the three libraries was based on a paired t-test, comparing tube panning R1 with chromato-panning R1.

## Authors' contributions

WN performed all practical experimental work. FP designed and produced the cryogel columns. IG participated in the experimental design. HP performed the statistical analysis. HD and BM supervised the work. The final manuscript was read and accepted by all co-authors.
